# A pilot fMRI study of the effect of stressful factors on the onset of depression in female patients

**DOI:** 10.1007/s11682-015-9382-8

**Published:** 2015-04-12

**Authors:** Gongying Li, Xiaoyan Ma, Haiman Bian, Xinhai Sun, Ning Zhai, Mengyuan Yao, Hongru Qu, Shengzhang Ji, Hongjun Tian, Chuanjun Zhuo

**Affiliations:** Department of Psychiatry, Jining Medical University, Jining City, Shandong Province 272013 Peoples Republic of China; Tianjin Academy of Traditional Chinese Medicine Affiliated Hospital, Hongqiao District, Tianjin, 300120 People’s of Republic of China; Tianjin 4th Centre Hospital, Hebei District, Tianjin, 300143 People’s Republic of China; Department of Magnetic Resonance Imaging, Affiliated Hospital of Jining Medical University, Jining City, Shandong Province 272000 People’s Republic of China; Tianjin Anning Hospital, No. 20 Yongping Road, Dongli District, Tianjin, 300300 People’s Republic of China; Department of Psychiatry Functional Imaging Laboratory, Tianjin Anding Hospital, Hexi District, Tianin, 300222 People’s Republic of China

**Keywords:** First major depressive episode, Stressful life events, Functional magnetic resonance imaging

## Abstract

The goal of this study was to observe the differences in brain activation under negative emotional picture stimuli in drug-naïve female patients with a first major depressive episode, comparing patients with and without stressful life experiences prior to the onset of depression. Using a 3.0 T magnetic resonance imaging (MRI) system, 18 patients who experienced stressful life events (SLEs) and 15 patients who did not experience SLEs were scanned under a task-fMRI paradigm designed to distinguish between negative and neutral neural responses to visual stimuli. SPM 8.0 software was used to process the fMRI data; the significantly activated brain regions were recorded and organized in the Montreal Neurological Institute (MNI) standard space. Upon stimulation with negative emotional pictures, depressed patients who had experienced SLEs showed significantly increased activation of the bilateral superior temporal gyrus, left middle temporal gyrus, left middle occipital gyrus, left medial frontal gyrus, right inferior frontal gyrus, bilateral precentral gyrus, bilateral postcentral gyrus, bilateral middle frontal gyrus, right precuneus, left paracentral lobule, bilateral thalamus, bilateral hippocampus, and left cerebellum when compared with depressed patients who did not experience SLEs.The brain regions that showed increased activation in depressed patients who experienced SLEs were primarily located in the neural circuits of the emotion processing system; this result likely indicates that these patients may have an increased negative cognitive bias in the perception, experience, and memory of negative emotional events, as well as their response to those events.

## Introduction

Negative stressful life events (NSLEs), such as bereavement, accidental disasters, and marital disruption, are closely related to the occurrence of major depressive disorders. A previous epidemiological study suggested that more than 90 % of depressed patients experienced stressful life events prior to the occurrence of the depressive disorder (Lueboonthavatchai 2009), indicating that stressful life events may be a factor that induces or accelerates the onset of major depressive symptoms in some patients. However, contradictory results also exist; another epidemiological study reported that some depressed patients did not experience any obvious stressful life events prior to the onset of their depressive symptoms (Kendler et al. 1999). According to previous reports, depression can be described as either endogenous depression or reactive depression depending on whether it was influenced by stress factors prior to its onset (Mizushima et al. 2013). However, studies on brain function and emotional processing bias between these two depression categories are lacking. Earlier studies showed that emotional processing bias is an important reason for the occurrence and maintenance of depression and may be a reflection of biological processing bias (Drevets et al. 2008; Gollan et al. 2008). Many functional magnetic resonance imaging (fMRI) studies have shown that the prefrontal cortex, anterior cingulate, insula, hypothalamus, amygdala, striatum, and hippocampus are involved in emotion formation and regulation. Abnormal brain activity in these regions is related to emotional processing bias (Davidson et al. 2003; Irwin et al. 2004; Mingtian et al. 2012; Perlman et al. 2012; Posse et al. 2003) . However, to the best of our knowledge, there is no study investigating the differences in brain activity related to the emotional processing bias between patients with endogenous and reactive depression.

Therefore, it is worthwhile to investigate whether brain functional activities related to emotional processing bias are different between first episode depression patients with stressful life events, which may represent the differences between patients with endogenous and reactive depression to some extent. Based on the results of previous studies, we hypothesized that reactive depression is likely to have specific brain functional abnormalities when compared with endogenous depression and that these specific functional abnormalities are likely related to SLEs experienced prior to the onset of depression. To this end, we plan to conduct a series studies to test this hypothesis. First, we conducted a pilot study to compare two groups of drug-naïve female patients experiencing their first major depressive episode using high field MRI. In this pilot study, our goal was to explore the specific functional abnormalities between patients who had experienced SLEs and those who had not, to provide the foundation for further large sample long-term follow-up studies.

## Methods

### Subjects

This study included inpatients and outpatients diagnosed with their first major depressive episode at Ankang Hospital in the Shandong Province of China between February 2011 and February 2013. ICD-10 criteria were used to diagnose the first major depressive episode (Organization 1992). Because male patients with depression in our sample population had a high likelihood of comorbid alcohol or nicotine abuse, we enrolled only female patients. All participants were right-handed, of Han Chinese ethnicity, and between 18 and 55 years of age. The eyesight or corrected visual acuity of participants was 1.0 or greater. All participants were diagnosed with their first major depressive episode and had never been treated with antidepressants, mood stabilizers, or antipsychotics prior to participation in the study. Patients were divided into two groups according to the information reported by patients and their relatives regarding whether the patient experienced negative stressful life events during the 6 months prior to the depressive episode. The influence of these SLEs on patients was quantitatively evaluated using the Life Event Scale (LES). We excluded patients who had a history of disturbances in consciousness greater than 5 min, nervous system diseases, other serious mental illness, substance abuse, serious physical disease, or endocrine disease. Pregnant and/or breast-feeding subjects, subjects who were previously enrolled in other research studies, and subjects who had contraindications to fMRI scans were also excluded. The Ethics Committee of Ankang Hospital approved this study. Written informed consent was obtained from all participants.

#### Clinical assessments

The 24-item Hamilton Depression Scale (Hamilton 1967) was used to evaluate depressive disorders in this study. This scale is the most widely used instrument for assessing adults with depressive symptoms; illness severity is reflected in the total score of this scale.

#### Quantitative evaluation of the influence of SLEs

The Life Event Scale (Yang and Zhang 1999) was used to evaluate the impact of life events and the severity of stress 6 months prior to the onset of the major depressive episode. There are 48 items included on the Life Event Scale, including assessments of family life, work/study, and social life.

### Study design and procedures

#### Emotional picture stimuli task design

This experiment adopted a block pattern design. Stimuli were composed of eight blocks: four stimulus blocks and four control blocks. The duration of each block was 30 s, with five pictures in each block and 6 s for each picture stimulus. Pictures were taken from the International Affective Picture System (IAPS, 2005 edition) (Lang et al. 2005) which is a set of standardized emotional stimuli. Twenty negative pictures with valence scores close to 1 (1.02 ± 0.013) and arousal scores close to 4.5 (4.42 ± 0.16), and twenty neutral pictures with valence scores close to 4.5 (4.48 ± 0.16) and arousal scores close to 4.5 (4.40 ± 0.15) were selected for this experiment. The block design was as follows: + − N-C-N-C-N-C-N-C (+ indicates rest, N indicates a neutral picture, and C indicates a negative picture). Subjects received alternate stimulation using neutral and negative pictures. They were given the following instructions before the experiment: “During the NMR scan, you will see some pictures. Please watch these pictures. Concentrate and do not think of other things. You might have some emotional experience when you view the pictures. Let them be freely released. Do not suppress or hide the emotions you experience.”

#### fMRI acquisition and parameter design

Imaging data were acquired using a 3.0 T Siemens system (Siemens Magnetom Trio Tim, Germany). The main scanning parameters were as follows: T1WI sequence: repetition time (TR) = 350 ms; echo time (TE) = 2.5 ms; slice thickness = 5.5 mm; gap = 1.1 m; matrix = 320 × 320; field of view (FOV) = 230 × 230 mm. T2WI sequence: TR = 6000 ms; TE = 93 ms; slice thickness = 5.5 mm; gap = 1.1 m; matrix = 320 × 320; FOV = 230 × 230 mm. T1WI and T2WI sequences were used to exclude subjects who had significant brain disease. Next, an echo planar imaging (EPI) sequence was adopted. Images were obtained using TR/TE = 3000 ms/30 ms, slice thickness = 3 mm, interval = 1 cm, flip angle = 90°, FOV = 200 × 200 mm, matrix = 64 × 64, voxel size = 3.1 × 3.1 × 3.0 mm, plies number = 36, and gap = 0.75 mm. For the EPI sequence, it took 4 min and 9 s to complete one test, including a 9 s pre-scan performed to obtain a stable signal. The pre-scan was not included in the data processing.

#### Experimental procedure design

To improve experimental compliance and ensure the completion quality of the stimuli task during the fMRI scan, a similar picture stimuli task training exercise was given to participants before the actual test. In doing so, the test content would therefore be fully understood by the subjects prior to beginning the actual test.

### fMRI data analyses

Image pre-processing and statistical analyses were conducted using statistical parametric mapping (SPM8; Wellcome Department, United Kingdom) and a random-effects model for group analyses. Data from each session were pre-processed, including slice-timing, realignment and normalization into a standard template (Montreal Neurological Institute, Canada). Smoothing was applied with a 4 × 4 × 4 mm, 3 full widths, at half-maximum isotropic Gaussian kernel. For the first-level analyses, a general linear model was established using subjects’ brain images after space preprocessing, making each scan a unit. The scan interval was 3 s. Neutral pictures and negative pictures were set as the two stimulation conditions. Ten scans were performed for every block so 80 MRI images were finally obtained for each patient. Each image was allocated a sequence number and input into SPM for analyses. For the model estimation, the parameters were evaluated and calculated using the restricted maximum likelihood method. The results reported were the activation in response to the different picture stimuli by contrast manager, showing the input contrast weights vector corresponding to the sequence set by the model. The contrast weights vector of the neutral pictures was 100. In the setup of SPM, the “contrast weights vector” in the “Contrast Manager” was set at −1 for neutral pictures and 1 for negative pictures. The activated brain areas under the “neutral pictures” setting were set as the baseline to illustrate the differences in activated brain areas in response to negative pictures. This study set the P-value threshold at 0.05; voxels were 0. Parameter files, including the spatial location of activation, were obtained after both types of picture stimuli. Second-level analyses were of factorial design; intra-group and inter-group analyses were performed first, with neutral pictures as baseline. One-sample t-tests were used to obtain average brain activation regions in response to the negative pictures for all of the patients. Based on above analysis, we then calculated the specific brain activation regions in response to the negative emotional pictures in the patients with and without SLEs. Two-sample t-tests were performed to determine the difference in brain activation regions between the two groups. The results were displayed by xjView software and the statistically significant (*P* < 0.05, AlphaSim corrected) regions were organized in the MNI standard space (x, y, z) and Brodmann’s area. The strength of the activated regions was also recorded (T used *t*-test statistics, greater T values indicate a greater strength of activation).

### Statistical analyses

Data were analyzed using SPSS 19.0 statistical analysis software. A *P*-value < 0.05 was considered statistically significant and all reported *P*-values are two-tailed. Continuous variables are presented as the mean ± standard deviation (SD).

## Results

### Social and demographic characteristics

In the current study, 23 subjects were evaluated, including 10 subjects who experienced SLEs and 13 subjects who had not experienced SLEs. There were no significant differences in age, and education level between the two groups. The quantity of SLEs was higher in the group that experienced SLEs compared with the group that did not experience SLEs (Table 1). Unfortunately, in the current study, all patients reported the stressful life events they experienced were negative events, such as divorce or unemployment.

**Table 1:** Socio-demographic and clinical characteristics of the participants

	SLEs (*n* = 10) Mean (SD)	Non-SLEs (*n* = 13) Mean (SD)	*t*	*P*
Age (years)	41.7 (9.26)	46.85 (13.89)	1.009	0.071
Duration of illness (months)	4.20 (3.73)	4.92 (3.30)	0.492	0.309
Education (years)	8.80 (4.18)	5.85 (5.55)	1.401	0.302
HAMD-24	49.50 (8.32)	45.92 (6.79)	1.137	0.356
LES-stimulus level	28.00 (9.03)	2.43 (1.92)	10.004	0.005

*Abbreviations*: *HAMD*-*24*, Hamilton depression scale, 24-item version; *LES*, life event scale

### fMRI data analyses

#### Average activation of brain regions stimulated by negative emotional pictures

**Fig. 1:**
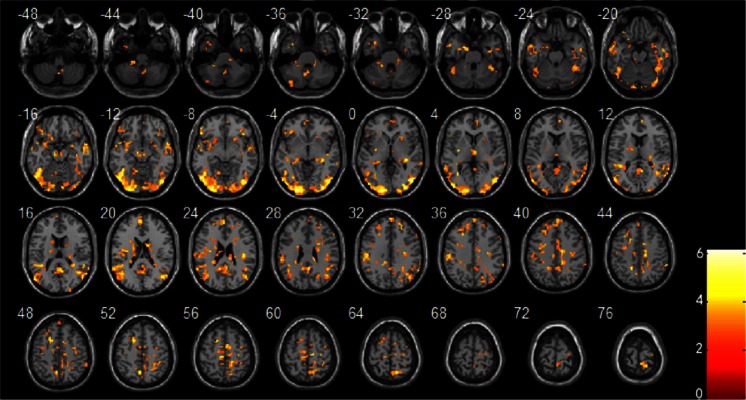
Brain regions with average increased activation in all patients with depression stimulated using negative emotional pictures compared with neutral emotional pictures. *P* < 0.05 and cluster size ≥ 19 (AlphaSim correction)

Table 2 and Fig. 1 show the average activation of brain regions in all of the patients stimulated by negative emotional pictures. These regions include the bilateral middle temporal gyrus, left inferior temporal gyrus, left inferior occipital gyrus, left superior occipital gyrus, bilateral lingual gyrus, bilateral fusiform gyrus, left medial superior frontal gyrus, right precuneus, bilateral angular gyrus, left cerebellum, and brainstem.

**Table 2:** Activated brain regions in all patients viewing negative pictures compared with neutral pictures

AAL brain area	Cluster size	MNI coordinate	*T* value	BA Zone
Cerebellum_9_R	44	−3 −60 −39	3.71	
Cerebellum_10_L	40	−18 −42 −33	4.14	
Temporal_Inf_L	31	−33 6 −33	4.52	38
Right Brainstem	19	12 −27 −42	3.59	
Cerebellum_Crus2_L	19	−36 −78 −36	3.66	
Bilateral Occipital lobe	3413	−51 −72 −6	6.26	18.31
Frontal_Inf_Orb_L	283	−33 −12 3	4.89	21.47
Temporal_Mid_L				
ParaHippocampal_L	36	−18 −3 −27	5.01	28
Temporal_Mid_R	104	57 6 −12	5.14	21
Midbrain	48	6 −18 −12	4.55	
Frontal_Inf_Orb_R	37	42 21 −6	4.70	11
Cerebellum_4_5_L	26	−12 −57 −9	4.68	19
Putamen_R	22	24 15 −9	3.45	
Frontal_Sup_Medial_R	51	9 54 9	5.73	10
Hippocampus_R	28	33 −30 −3	4.79	
Thalamus_R	31	3 −12 3	4.73	
Caudate_L	77	−18 −12 18	4.21	
Caudate_R	69	15 −3 18	6.01	
Frontal_Sup_Medial_L	217	3 57 33	5.03	9.10
Frontal_Mid_L				
SupraMarginal_L	301	−57 −30 30	5.57	40
Frontal_Sup_L				
Cingulum_Mid_R	22	15 −33 27	3.99	
Precentral_R	77	42 −21 24	4.77	4
Postcentral_R				
Frontal_Mid_R	41	24 42 33	4.29	9
Occipital_Sup_R	22	21 −69 30	4.69	19
SupraMarginal_R	44	63 −42 45	4.37	40
Cingulum_Mid_L	20	−12 −39 30	4.30	31
Frontal_Inf_Oper_R	19	57 24 30	3.60	9
Precuneus_L	23	−15 −48 42	6.05	7
Cingulum_Mid_L	44	−6 −24 42	3.44	6
Precuneus_L	29	−3 −60 51	4.70	7
Precentral_R	25	33 −6 48	3.94	6
Precentral_R	29	30 −21 60	4.23	6
Paracentral_Lobule_L	27	−6 −27 57	5.47	6

*AAL*, anatomical automatic labeling; *R*, right; *L*, left; *Inf*, inferior; *Mid*, middle; *Sup*, superior; *Oper*, operculum; *Orb*, orbitalis; *MNI*, montreal neurological institute

*P* < 0.05 and cluster size ≥ 19 (AlphaSim correction)

#### Comparison between the two groups upon stimulation with negative emotional pictures

**Fig. 2:**
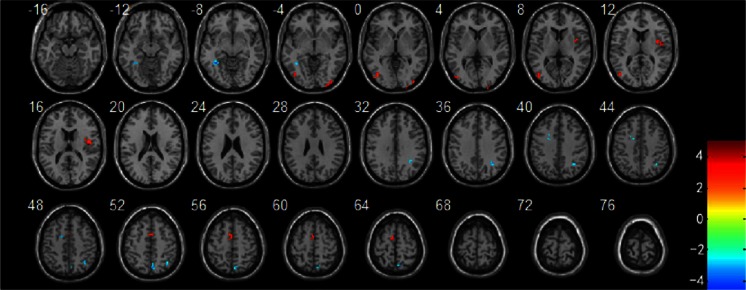
Brain regions with increased activation in patients with SLEs compared to patients without SLEs when stimulated using negative emotional pictures compared with neutral emotional pictures. *P* < 0.05 and cluster size ≥ 18 (AlphaSim correction)

Compared with patients who had not experienced SLEs, the bilateral superior temporal gyrus, left middle temporal gyrus, left middle occipital gyrus, left medial frontal gyrus, right inferior frontal gyrus, bilateral precentral gyrus, bilateral postcentral gyrus, bilateral middle frontal gyrus, right precuneus, left paracentral lobule, bilateral thalamus, bilateral hippocampal, and left cerebellum showed significantly increased activation in the patients who had experienced SLEs upon negative emotional picture stimulation (Table 3 and Fig. 2). There were no brain regions noted with decreased activation in the SLEs group.

**Table 3:** Different activated brain regions in patients with SLEs compared to patients without SLEs when stimulated by negative emotional pictures

AAL brain area	Cluster size	MNI coordinate	*T* value	BA Zoning
Fusiform_L	19	−36 −48 −9	−4.49	19
Occipital_Mid_R	24	30 −96 −3	3.80	19
Occipital_Mid_L	32	−45 −81 6	3.99	19
Rolandic_Oper_R	28	48 −3 12	5.08	13
Angular_R	25	36 −57 48	−3.57	40
Frontal_Sup_L	18	−21 6 42	−3.32	9
Precuneus_R	39	6 −69 51	−4.04	7
Supplementary_Motor_Area_L	33	−6 −3 63	3.49	6

*AAL*, anatomical automatic labeling; *R*, right; *L*, left; *Mid*, middle; *Sup*, superior; *Oper*, operculum; *MNI*, montreal neurological institute

*P* < 0.05 and cluster size ≥ 19 (AlphaSim correction)

## Discussion

To the best of our knowledge, this is the first fMRI study to examine differences in the activation of brain regions upon stimulation with negative emotional pictures between two different groups of drug-naïve female patients during their first major depressive episode, who were divided according to their experience of SLEs.

In this study, when patients were stimulated with negative emotional pictures, the left medial frontal gyrus, right inferior frontal gyrus, bilateral precentral gyrus, and bilateral middle frontal gyrus showed significantly increased activation in patients who had experienced SLEs compared with patients who had not experienced SLEs. These brain regions belong to the area of the prefrontal lobe. Several studies have reported that the prefrontal lobe plays a key role in processing emotional information. For example, the prefrontal lobe participates in the cognitive assessment of stimuli during emotional task processing (Ongur et al. 1998). Increased activation in the inferior frontal gyrus (van Wingen et al. 2011), the left dorsolateral prefrontal cortex, rostral anterior cingulate, left parietal cortex, caudate, right amygdala (Dillon and Pizzagalli 2013) and right dorsolateral prefrontal cortex (Samson et al. 2011) were also found after negative emotional stimuli. Lemogne et al. reported that the dorsal medial prefrontal cortex may play a key role in integrating increased attention to negative stimuli (Lemogne et al. 2011). Collectively, these results indicate that patients with abnormal prefrontal lobe activation may be more sensitive to the perception and memory of negative emotional stimuli. This idea is consistent with the negative cognitive theory of depression, which proposes that negative stimulation is an important factor in the onset and maintenance of depression (van Wingen et al. 2011).

However, the results above compared differences in the activation of brain regions between depressed patients and healthy controls. These studies did not compare the differences between patients with endogenous and reactive depression; therefore, our results complete this knowledge gap to a certain extent. Our results showed stronger brain activation in the prefrontal lobe regions of patients with depression who experienced SLEs, indicating that these patients may be more sensitive to negative emotional stimuli when compared with patients who have not experienced SLEs. These data suggest that in the SLEs group, it is easier to perceive and remember negative emotional stimuli and easier to be influenced by negative stressful life events.

The occipital cortex belongs to the visual perception region; the visual cortex participates in external stimuli perception and transfers this information to the brain regions that relate to emotion processing and response (Adolphs 2001). The present study showed that visual cortex activity (specifically, the left middle occipital gyrus) was significantly increased in patients with depression who experienced SLEs when stimulated with negative emotional pictures. This finding indicates that these patients are more likely to perceive negative visual information compared to patients who have not experienced SLEs.

The temporal lobe participates in the processing of emotional experiences. Narumoto et al. reported that the superior temporal gyrus and the adjacent cerebral cortex play important roles in the processing of information related to individual communication (Narumoto et al. 2001). Chen et al. found increased activation of the temporal lobe regions in depressed patients during the processing of negative emotional experiences (Chen et al. 2006). The present study showed increased activation of the bilateral superior temporal gyrus and left middle temporal gyrus in patients with depression who had experienced SLEs during the processing of negative emotional picture stimuli. These data indicate that patients who have experienced SLEs are more inclined to experience negative emotions.

The precuneus is a region of the brain network that involves visuospatial information processing and participates in processing the perception of negative emotions. Abnormal activation of the precuneus may reflect an increasing sensitivity to the perception of negative emotions, thus making an individual more inclined to focus on the perception of negative emotions (Halari et al. 2009). The findings of the present study are consistent with previous reports, which indicate that the right precuneus is significantly activated in patients with depression who have experienced SLEs upon stimulation with negative emotional pictures.

The cerebellum is the relay station for emotional pathways in the limbic system (the frontal lobe, parietal lobe, and temporal lobe) and is also involved in the expression of negative emotions. Groenewold et al. showed increased activation in the right side of the cerebellum upon negative emotional picture stimulation, suggesting that emotional pathway activation is abnormal in depressed patients (Groenewold et al. 2013). The present study also found increased cerebellar activation in patients with depression who had experienced SLEs. However, conflicting results exist; some studies have reported that the cerebellum has a nonspecific response to different stimuli. These findings indicate that the definitive role of the cerebellum in the activation of emotional processing remains unclear and that further studies are required to clarify its distinct role (Turner et al. 2007). The thalamus is involved in the perception and regulation of emotions (Critchley et al. 2000; Drevets 2000; LaBar et al. 2003; Malhi et al. 2004). Our study also found increased activation in the bilateral thalamus when patients with depression who had experienced SLEs were stimulated with negative emotional pictures. This result indicates that the regulation bias and perception of negative emotions may be more pronounced in these patients compared to those who have not experienced SLEs.

The hippocampus plays an important role in the experience and regulation of emotions, especially in the background regulation of emotion and behavior. It is also involved in the memory retrieval processes of negative emotions (Allen et al. 2012). The present study showed increased activation in the bilateral hippocampus of patients stimulated by negative pictures, suggesting that these patients may be more likely to retrieve negative emotional memories of negative life events.

There are some limitations to this study. First, we only evaluated female patients. This decision was based on the higher prevalence of depression in women (Weissman et al. 1996). Moreover, male patients with depression in our sample population often have comorbidities or a history of smoking and excessive alcohol consumption (Burns and Teesson 2002), which would influence the fMRI results. Male patients experiencing major depressive disorder are also more likely to show agitation, aggression, and antisocial behavior (Kornstein et al. 2000). These behaviors make it difficult to complete fMRI scans during the negative emotional picture stimulation task. A second limitation is that we only compared brain activity differences in patients experiencing a major depressive episode. Due to the limited number of healthy controls, we could not compare differences in brain activity upon stimulation with negative emotional pictures among: patients with depression who had experienced SLEs, patients with depression who had not experienced SLEs, healthy controls who had experienced SLEs, and healthy controls who had not experienced SLEs. Therefore, our results are not sufficient enough to illustrate the neural mechanism(s) of SLEs related to the onset of a major depressive episode. Third, fMRI data in the present study were compared between patients after being diagnosed with their first major depressive episode. Therefore, the data do not explain the effect of SLEs on the onset of depression. By collecting fMRI data during the early stages after an SLE experience and at the time of depression onset, an analysis of the differences between these two time points may be helpful to explore the neural mechanisms of SLEs on the onset of depression. Fourth, considering the interactions between genes and stress, a long-term follow-up study with a large sample size that combines imaging techniques with genetic techniques is needed to explore the pathological mechanisms of SLEs on the onset of depression. In this study, we followed participants for only 12 months. Therefore, we cannot verify if any of our patients potentially had bipolar disorder. A longer follow-up period would be required to make this determination.

Well-controlled studies with large sample sizes are required to identify the potential neural mechanisms of SLEs on the onset of major depressive episodes. Similarly, long-term follow-up studies are needed to investigate the relationship between the therapeutic effects and early changes in functional brain imaging findings between the two different categories of patients. It would be helpful to explore predictive functional neuroimaging markers to optimize the clinical treatment of depression.

In conclusion, the present study found that drug-naïve female patients with their first major depressive episode who had experienced SLEs prior to the onset of depression had increased activation of several brain regions involved in emotional perception, memory, evaluation, regulation, experience, and expression. The results of our study indicate that patients with depression who experienced SLEs prior to the onset of depression may be more likely to perceive and experience negative emotional cues, resulting in the generation and expression of negative emotions; these findings are supported by the theory of negative cognition in depression (Beck 1964).
